# The role of multimodality imaging in diffuse pelvicoabdominal plexiform neurofibroma: A rare case report

**DOI:** 10.1016/j.radcr.2024.08.037

**Published:** 2024-09-07

**Authors:** Andi Ahmad Thoriq Pratama, M. Hidayat Surya Atmaja

**Affiliations:** a Department of Radiology, Dr. Soetomo General Academic Hospital, Surabaya, Indonesia; b Department of Radiology, Faculty of Medicine - Universitas Airlangga, Surabaya, Indonesia

**Keywords:** Plexiform neurofibroma, Multimodality imaging, Neurofibromatosis, Ultrasound, CT, MRI

## Abstract

Pelvicoabdominal plexiform neurofibroma is a rare and complicated form of type 1 neurofibromatosis (NF1), distinguished by developing benign nerve sheath tumors in the pelvis and abdomen. A male patient, aged 26, came to our center with dysuria, abdominal bloating, rectal mucosa prolapses, and trouble walking and moving legs. Physical examination revealed a palpable mass of solid consistency fixed in the pelvic cavity to the abdominal cavity. A large and extensive mass in the pelvic to the abdominal region can be evaluated with multimodality radiological imaging, including ultrasound, computed tomography, and magnetic resonance imaging. Imaging is crucial for diagnosis, evaluation of extension, and early detection of potential malignant transformation in these patients. The patient was scheduled for palliative surgical resection due to the extensive mass; however, he did not survive while waiting for the operation. Pathology examination and immunohistochemical staining revealed positive S-100 protein, indicating the neural crest originate lesion. We report the clinical and radiological features of plexiform neurofibroma in a young male patient, confirmed by pathology examination.

## Introduction

Type 1 neurofibromatosis (NF 1) and type 2 neurofibromatosis (NF 2) are separate genetic conditions that involve the development of noncancerous tumors known as neurofibromas. Although there are certain similarities between them, they exhibit distinct clinical characteristics and genetic compositions. It is caused by a mutation in the NF 1 gene located on chromosome 17q11.2 and is inherited in an autosomal-dominant manner. The prevalence of NF 1 is approximately 1 in 4000-5000, making it one of the most prevalent hereditary multi-tumor syndromes. The primary features of NF 1 involve the central nervous system (CNS), skin, and bone, with clinical manifestations consisting of bone lesions, including sphenoid dysplasia, axillary or inguinal freckling, neurofibromas or plexiform neurofibromas, optic pathway gliomas, Lisch nodules, and café-au-lait macules [1].

Numerous additional symptoms of NF 1 include low-grade gliomas, vascular dysplasia, various abdominopelvic neoplasms, and interstitial lung disease. NF 2 can present as ependymomas, meningiomas, and schwannomas [1]. NF 1 has various types, classified into 2 subtypes: localized and plexiform [2]. Plexiform neurofibroma is a poorly circumscribed and locally invasive tumor mass that extends along the length of a nerve trunk, growing around distorted nerve fascicules, and may spread along adjacent nervous rami, muscles, and skin. These lesions may grow superficially or deeply and entail high morbidity as they grow progressively, often producing disfigurement [3]. These tumors are at significant risk of developing a malignancy, especially in the nervous system [4]. Pelvicoabdominal neurofibroma is a rare disease with around 60 reported cases in the literature and only a few involving the prostate [4]. We report a unique case of pelvicoabdominal neurofibromatosis affecting the prostate, highlighting the significance of multimodality imaging in its diagnosis.

## Case presentation

**Fig. 1:**
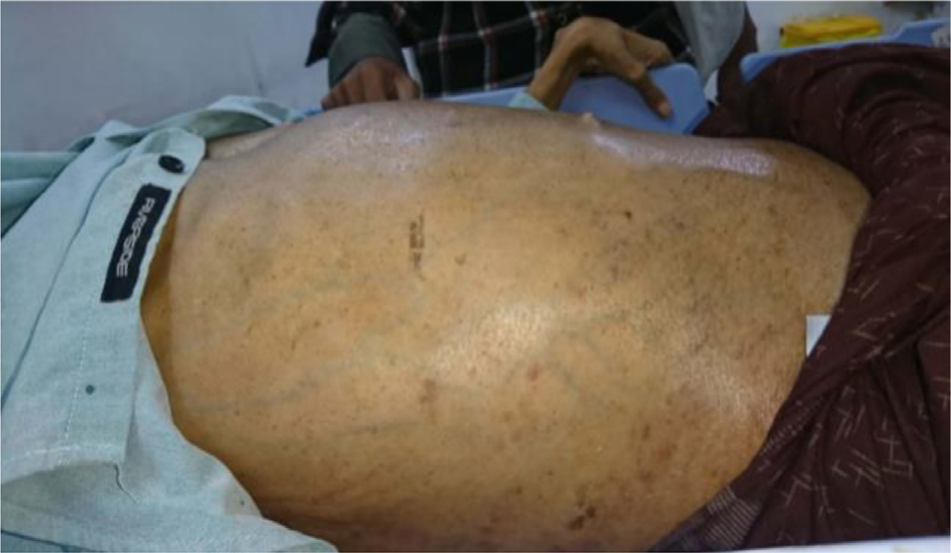
The abdomen appears bloated with several café-au-lait macules scattered in the abdominal region.

A 26-year-old male came to the hospital with dysuria and abdominal distension persisting for 5 years, along with rectal mucosa prolapse. In the past year, he experienced impaired mobility and struggled with walking and leg movement. No prior medical record of urinary tract infection was recorded. On physical examination, we observed anemia and multiple café au lait macules in the abdominal region (shown in Fig. 1). The laboratory test showed a normal prostate-specific antigen (PSA) (1.74 ng/mL).

**Fig. 2:**
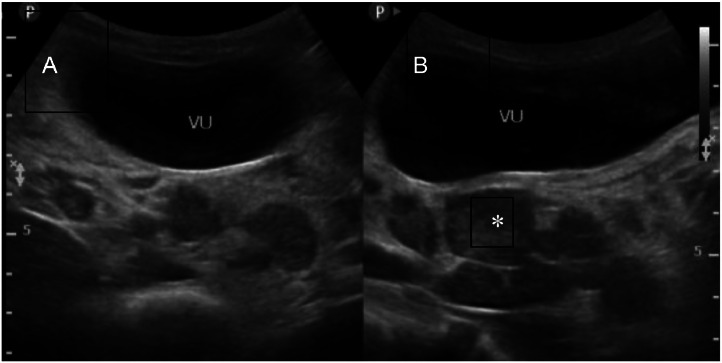
Abdominal ultrasound, transverse (A) and longitudinal (B) sections revealed the presence of multiple well-defined oval hypoechoic masses in the abdominal cavity (*).

**Fig. 3:**
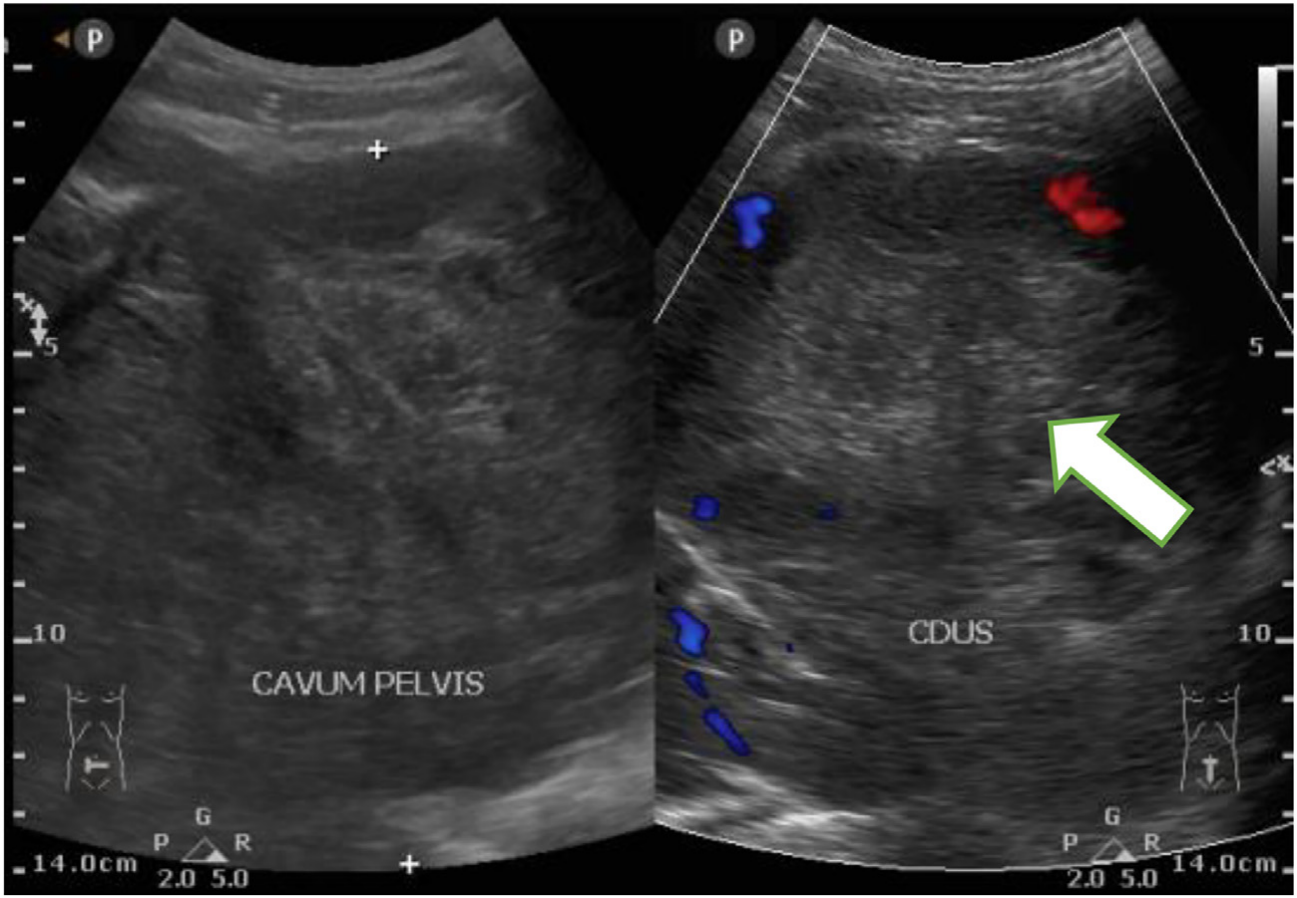
On abdominal ultrasound examination, transverse view showed a large hetero-echoic mass filling the abdominal cavity and pelvic cavity, which on Doppler appeared hypovascular (arrow).

Ultrasound examination showed multiple hypoechoic lesions with oval shapes and well-defined borders from the abdominal cavity to the pelvic cavity, along with a target sign with a hyperechoic center and hypoechoic edges. In the pelvic area, a large heteroechoic mass was found in the pelvic and abdominal cavity, which appeared hypovascular on Doppler examination (Figs. 2 and 3).

**Fig. 4:**
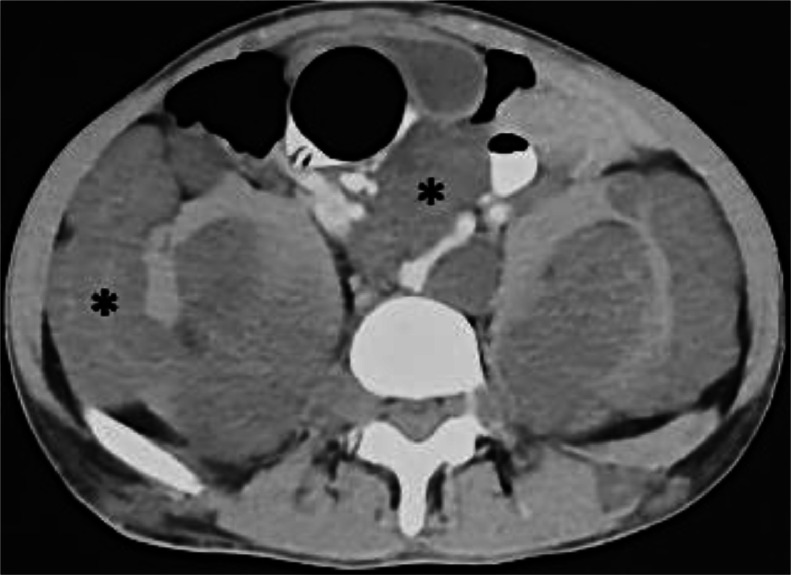
Axial section of the abdominal CT scan revealed multiple masses with low density compared with muscle at intraperitoneal and extraperitoneal (asterisk).

**Fig. 5:**
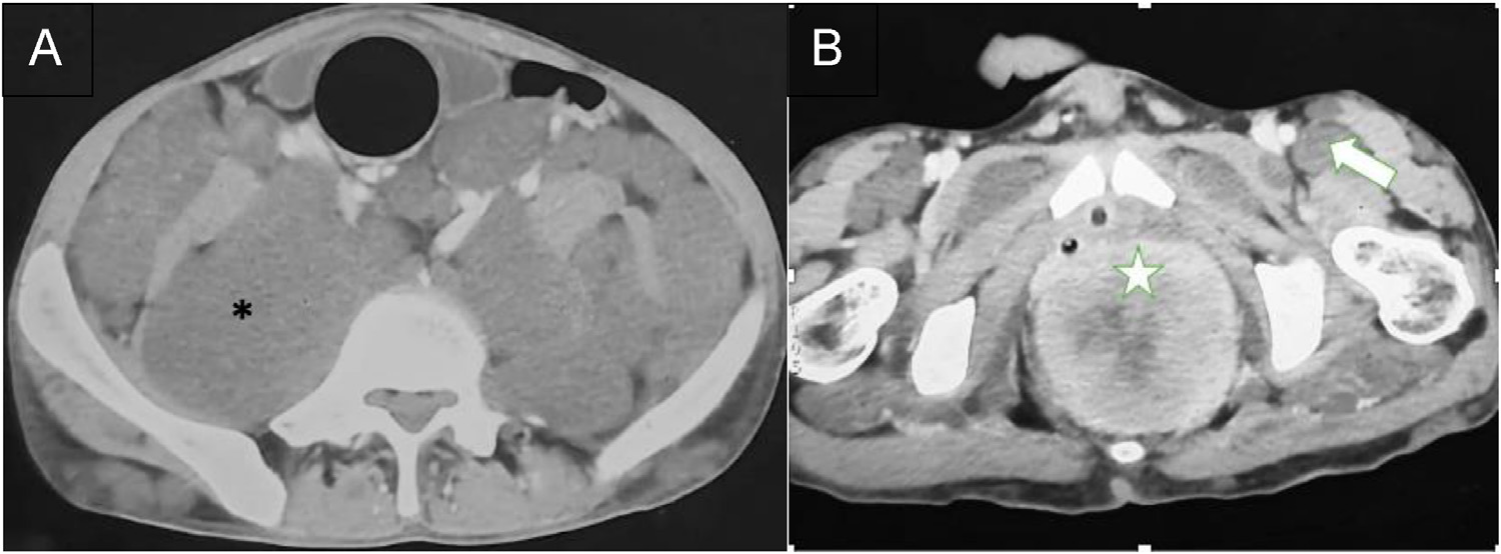
Contrast-enhanced axial section of abdominal CT scan (A,B) depicted a large heterogenous mass (B, white star) that seemed to arise from the prostate and several numerous small low attenuation masses in intra-extraperitoneal (A, asterisk) and also along the inter-intramuscular (B, white arrow).

Computed tomography (CT) imaging showed multiple bilateral, symmetrical, and lobulated masses with low attenuation in the abdominal and pelvic cavity, filling the intraperitoneal, extraperitoneal, intramuscular, and intermuscular spaces with vascular encasement (Fig. 4). Subsequently, a sizable tumor in the prostate was also noted, displaying varied contrast enhancement. The tumor extended into the abdominal cavity and pushed the bladder to the anterosuperior (Fig. 5).

**Fig. 6:**
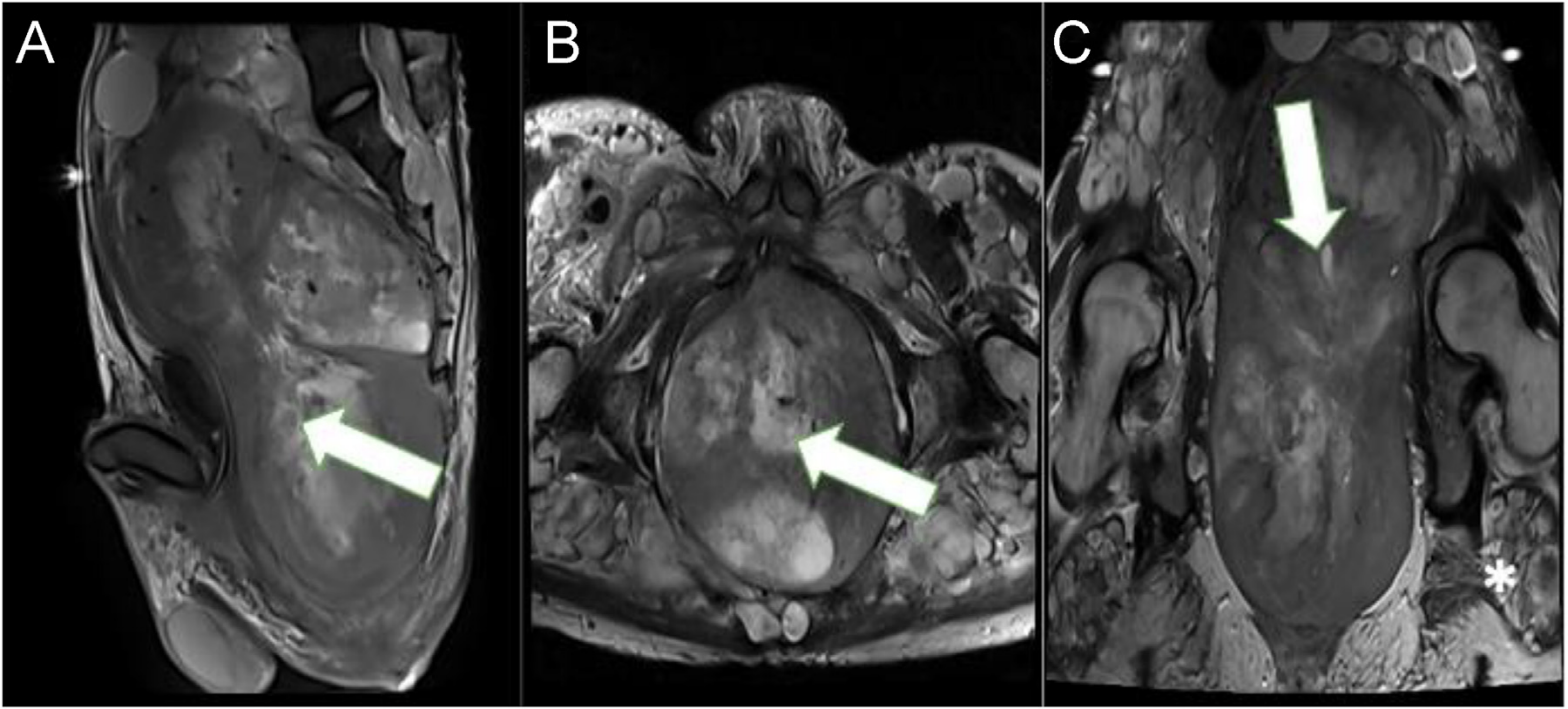
(A) Sagittal, (B) axial, and (C) coronal view of T2-weighted abdominopelvic MRI = revealed heterogenous mass from the prostate extending to the abdominal cavity (A-C, white arrow). There were masses with target signs in the presacral space, along with intra and intermuscular (*).

**Fig. 7:**
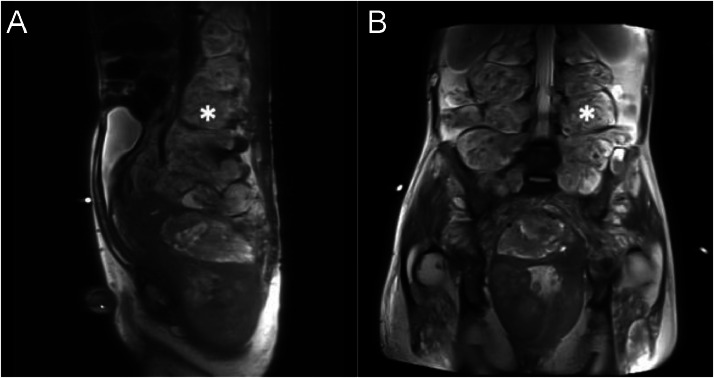
(A) Sagittal and (B) coronal projection of T2-weighted MRI showed the multilevel lobulated mass with a hyperintense signal with the target sign on T2WI extending along right and left lumbar neural foramina (asterisk).

**Fig. 8:**
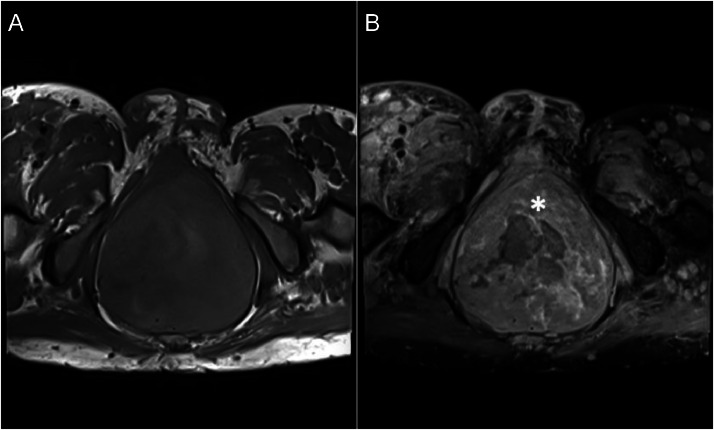
MRI axial projection showed noncontrast T1-weighted imaging (A) showed a giant mass with solid and necrotic area arising from the prostate, measuring approximately 9.2 × 19.3 × 9.6 cm, extending to the abdominal cavity, which, on gadolinium contrast-enhanced T1-weighted fat-suppressed imaging (B) exhibited heterogenous contrast enhancement on solid components (asterisk).

Magnetic resonance imaging (MRI) findings showed a complex mass in the prostate extended into the pelvic cavity, extensive enlargement of the fusiform mass at the lumbosacral nerve root, and multiple plexiform lesions in the intramuscular and intra-extraperitoneal areas that compressed the surrounding organs, especially the bladder to the anterosuperior (Fig. 6, Fig. 7). The tumor was heterogeneous on T2-weighted imaging, with a target sign appearance. This sign, which is unique to plexiform neurofibromas, is caused by a central fibro-collagenous core (T2-hypointense) surrounded by myxomatous tissue (T2-hyperintense) and low signal intensity on T1-weighted imaging. A diffusion-weighted imaging (DWI) of the mass also showed a restricted diffusion area. The prostate mass was presented as a solid mass with a necrotic area, which, on contrast administration, showed heterogeneous contrast enhancement (Fig. 8).

**Fig. 9:**
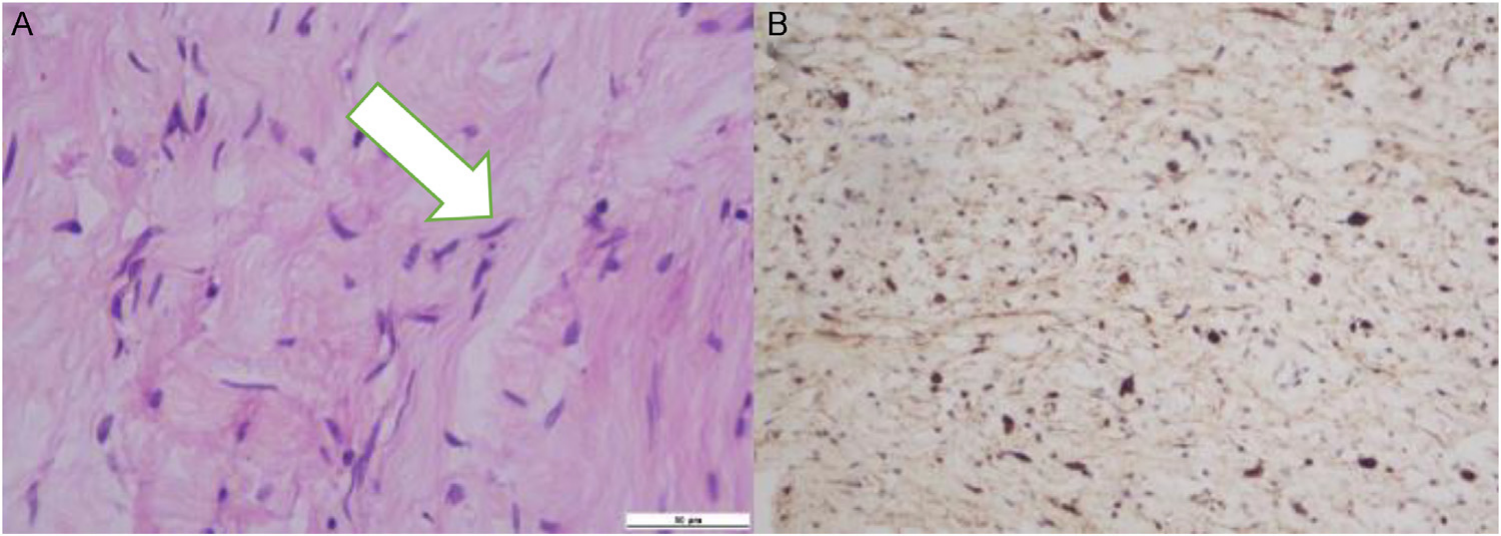
Pathology examination of the abdominal mass displayed nuclei with a wavy appearance (white arrow), smooth chromatin without signs of malignancy (A, x400 magnification, H&E stain). (B) Immunostaining expressed positive staining of tumor cells for S100 antibody.

The patient underwent an open biopsy, resulting in neurofibroma, confirmed by immunohistochemical staining positive for S-100 protein, indicating cells of neural crest origin, and a negative stain for vascular, muscular, and epithelial markers. A diagnosis of neurofibroma was established (Fig. 9). There was neither nuclear pleomorphism nor mitotic figures to indicate malignancy. Nevertheless, the treatment options for our patient were severely limited due to the extensive intraabdominal and intra-thecal involvement. The patient was then scheduled for palliative surgical resection due to the extensive mass; however, he did not survive while waiting for surgery.

## Discussion

Neurofibromas are neoplasms that originate from Schwann cells, are located in any part of the body, and are predominantly observed in the skin. Neurofibromatosis is a hereditary condition that can be identified by specific diagnostic criteria, such as the presence of one or multiple neurofibromas, Lisch nodules in the iris, multiple café-au-lait spots, bony changes, optic nerve glioma, and other symptoms [4]. A plexiform neurofibroma, a less prevalent type of benign neurofibroma, is a tumor that occurs specifically in individuals with NF I. It occurs due to the uncontrolled growth of all neural components in a peripheral nerve. The term “plexiform” describes an intricate network of interconnected or intertwined blood vessels or nerves, as observed in this particular condition. The term “plexiform” originates from the Latin verb “plex,” which signifies the act of weaving or intertwining [5].

Pelvic neurofibroma can present either standalone or as a component of the multiorgan abnormality known as NF-1 [3]. Genitourinary involvement is infrequent, with a mere 60 documented cases; tumors originating from the nerve plexus surrounding the bladder trigone can impact the prostate, seminal vesicle, and urethra in males [6]. The clinical presentations of this condition, including frequency, recurrent urinary tract infections, and urgency, are not uncommon [7]. While infrequent, malignant degenerations can occur. Diagnosing a pelvicoabdominal neurofibroma is easier when associated with another syndrome. However, in isolated cases, the differential diagnosis for a mass in the base of the bladder should include rhabdomyosarcoma, paraganglioma, ganglioneuroma, and leiomyosarcoma.

Plexiform neurofibroma appears as thickened firm masses or nodules that may deeply infiltrate structures, causing deformation and dysfunction. At MRI, they manifest as multinodular confluent masses with mass effect on surrounding structures and multiple target signs [1] that have the potential to transform into malignant peripheral nerve sheath tumors (MPNST). MPNST has a poor prognosis due to metastases to the brain, lung, liver, soft tissue, bone, regional lymph nodes, skin, and retroperitoneum. The estimated lifetime risk of neurofibromatosis undergoing malignant transformation is 4.6% [8]. Hence, a patient with plexiform neurofibroma is 20 times more likely to have MPNST than one without plexiform neurofibroma [9]. NF1-associated MPNST typically begins as plexiform neurofibroma, with a prevalence in the general population of 0.001%. Therefore, patients with NF1 have a significantly higher likelihood of developing MPNST, with a risk of 4,600 times greater than those without NF1 [8].

We conducted a comprehensive assessment of our patient utilizing ultrasound (US), MRI, and CT scans. Imaging is essential for various reasons: it aids in assessing the degree of involvement and its effects on nearby structures, detects associated abnormalities, and, most importantly, malignant transformation. Ultrasound is frequently ordered to assess palpable soft-tissue masses. The differential diagnosis depends upon the characteristics of the tumor component. Peripheral nerve sheath tumors can display a range of sonographic features, with predominantly appearing as well-defined oval-shaped hypoechoic masses connected to peripheral nerves. These masses frequently exhibit a target-like appearance on ultrasound, characterized by a hyperechoic center and a darker outer edge with increased posterior enhancement behind it. On color Doppler imaging (CDU), these structures usually appear to have reduced blood flow, although there may be instances where internal flow is observed [10]. It is crucial to distinguish these tumors from vascular malformations and infections such as abscesses. Vascular malformations can be ruled out with CDU, for the tumor doesn't show increased blood flow upon compression and release. The tumor is without internal moving echoes, which are characteristic of an abscess. Nevertheless, it is crucial to acknowledge that ultrasound lacks the ability to consistently differentiate between benign and malignant lesions [5,11].

Neurofibromatosis is characterized by a soft tissue mass with low attenuation on CT scans. This is caused by the presence of myelin-lipid content, high water content, and fat trapped in the endoneurial myxoid tissue [11]. Our patient exhibited similar low-attenuation lesions in the intraperitoneal and extraperitoneal regions, with no bone involvement. A CT scan provides the advantage of assessing bone involvement. In suspected malignant lesions, the CT scan reveals heterogeneity with centrally noted necrosis, indistinct margins, and irregular nodular peripheral enhancement [11].

MRI is still the best way to diagnose neurofibromatosis (NF) with characteristic of a “target sign” on T2-weighted images, a hyperintense peripheral rim and a hypointense central fibrous component, as well as central enhancement. In cases of plexiform neurofibroma, MRI shows a multinodular confluent mass with multiple target signs and a mass effect on nearby structures. It is critical to obtain a definitive soft tissue diagnosis to rule out malignancy, as plexiform neurofibroma has the potential to become malignant [12]. Since the target sign was absent in several neurofibromas, it was not a reliable indicator of malignancy [13]. MRI is essential for differentiating between neurofibroma and malignant peripheral nerve sheath tumor (MPNST). This is accomplished by evaluating the increased largest dimension of the mass, the swelling around the lesion, the presence of fluid-filled sacs within the tumor, and the peripheral enhancement pattern. The presence of 2 to 4 of these features suggests malignancy, with specificity and sensitivity of 90% and 61%, respectively [13]. In this patient, the large mass arising from the prostate had features not typically seen in patients with generalized neurofibroma. However, there was no peripheral enhancement pattern, no perilesional edema-like zone, and the margin was still well-defined, indicating that the mass in the prostate had not undergone malignant transformation [14].

The final diagnosis is determined based on the histopathology results. We performed an open biopsy on our patient with result spindle cells showed primarily positive for the S-100 protein on immunostains, without malignant transformation features existed. Neurofibromas are benign tumors that form in the peripheral nerves. They consist of Schwann cells and nonneoplastic peripheral nerve components, including fibroblasts, perineurial cells, blood vessels, axons, and mast cells[15]. The immunohistochemical staining for S-100 protein is more prominent and uniform in schwannomas compared to neurofibromas. Malignant peripheral nerve sheath tumor (MPNST) exhibits sporadic expression of S100, which is detected in only 50% to 60% of the instances. Furthermore, most superior-quality specimens yield negative results for this protein [15,13].

Treating plexiform neurofibromas requires a multidisciplinary approach, usually involving surgery, aiming to resect the deformed mass and cancerous tissue when malignant transformation occurs. In addition, complete resection is often challenging due to the extensive growth of the tumors, invasion of the surrounding tissues, and the tendency of re-growth after surgery. In several studies in patients who did not undergo surgery, might consume medication such as interferon and mitogen-activated protein kinase (MEKi), such as selumetinib, that may reduce the volume and symptoms caused by the mass [16,17]. Considering these factors and the avoidance of ionizing radiation, MRI is the best modality for patient monitoring [18]. Malignant transformation of the neurofibromatosis most effective primary treatment is complete surgical resection. Additional therapy, such as radiotherapy or neoadjuvant therapy, including chemotherapy, is optional for unresectable cases or to reduce the risk of recurrences. However, the prognosis remains unpredictable due to the high risk of disease progression and its variable manifestation [16,19].

## Conclusion

Plexiform neurofibroma is a distinct variety of nerve tumors that typically manifests in individuals diagnosed with NF1. This neoplasm arises from the nerve sheath and is distinguished by numerous lobulated masses and a distinctive imaging characteristic known as the target sign. They have the ability to impact both the subcutaneous tissue and the skin at the same time while also penetrating deeper structures. When assessed using ultrasound, CT, and MRI, these tumors typically exhibit fat and fluid content as a result of the myelin content in the nerve sheath. Radiological imaging is paramount in confirming the diagnosis, identifying affected structures, ruling out other conditions, and detecting potential malignant transformation, which can help determine the patient's treatment options.

## Patient consent

Written informed conscent was obtained from the patient for the publication of this case report.

## References

[1] Wang MX, Dillman JR, Guccione J, Habiba A, Maher M, Kamel S (2022). Neurofibromatosis from head to toe: what the radiologist needs to know. Radiographics.

[2] Bergqvist C, Servy A, Valeyrie-Allanore L, Ferkal S, Combemale P, Wolkenstein P (2020). Neurofibromatosis 1 French national guidelines based on an extensive literature review since 1966. Orphanet J Rare Dis.

[3] Bouimetarhan L, Bellamlih H, En-Nafaa I, El Fenni J, Amil T, Radouane B. (2018). Plexiform cervical neurofibroma: about a case. Pan African Med J.

[4] Jana M, Gamanagatti S, Kumar R, Aggarwala S. (2011). Pelvic neurofibroma arising from prostate in a case of neurofibromatosis-1. Indian J Urol.

[5] Grover DSB, Kundra DR, Grover DH, Gupta DV, Gupta DR. (2021). Imaging diagnosis of plexiform neurofibroma- unravelling the confounding features: a report of two cases. Radiol Case Rep.

[6] Wong-You-Cheong JJ, Woodward PJ, Manning MA, Sesterhenn IA. (2006). From the archives of the AFIP: neoplasms of the urinary bladder: radiologic-pathologic correlation. Radiographics.

[7] Pascual-Castroviejo I, Lopez-Pereira P, Savasta S, Lopez-Gutierrez JC, Lago CM, Cisternino M. (2008). Neurofibromatosis type 1 with external genitalia involvement. Presentation of 4 patients. J Pediatr Surg.

[8] Ducatman BS, Scheithauer BW, Piepgras DG, Reiman HM, Ilstrup DM. (1986). Malignant peripheral nerve sheath tumors. A clinicopathologic study of 120 cases. Cancer.

[9] Tucker T, Wolkenstein; P, Revuz; J, Zeller; J, Friedman JM. Association between benign and malignant peripheral nerve sheath tumors in NF1. 2005. 10.1212/01.wnl.0000168830.79997.13 16043787

[10] Reynolds DL, Jacobson JA, Inampudi P, Jamadar DA, Ebrahim FS, Hayes CW. (Mar 2004). Sonographic characteristics of peripheral nerve sheath tumors. Am J Roentgenol.

[11] Gosein M, Ameeral A, Banfield R, Mosodeen M. (2013). Plexiform neurofibroma of the wrist: imaging features and when to suspect malignancy. Case Rep Radiol.

[12] Zulfiqar M, Lin M, Ratkowski K, Gagnon MH, Menias C, Siegel CL. (2021). Imaging features of neurofibromatosis type 1 in the abdomen and pelvis. Am J Roentgenol.

[13] Wasa J, Nishida Y, Tsukushi S, Shido Y, Sugiura H, Nakashima H (2010). MRI features in the differentiation of malignant peripheral nerve sheath tumors and neurofibromas. Am J Roentgenol.

[14] Rotili A, De Maria F, Di Venosa B, Ghioni M, Pizzamiglio M, Cassano E (2018). Solitary breast neurofibroma: imaging aspects. Ecancermedicalscience.

[15] Guedes-Corrêa J, Cardoso R. (2018). Immunohistochemical markers for schwannomas, neurofibromas and malignant peripheral nerve sheath tumors: what can the recent literature tell us?. Brazilian Neurosurg.

[16] Dare AJ, Gupta AA, Thipphavong S, Miettinen M, Gladdy RA. (2020). Abdominal neoplastic manifestations of neurofibromatosis type 1. Neurooncol Adv.

[17] Fisher MJ, Blakeley JO, Weiss BD, Dombi E, Ahlawat S, Akshintala S (2022). Management of neurofibromatosis type 1-associated plexiform neurofibromas. Neuro-Oncology.

[18] Niku SD, Mattrey RF, Kalota SJ, Schmidt JD (1995). MRI of pelvic neurofibromatosis. Abdom Imaging.

[19] Bin Abdul Halim WMA, Bin Mat Hassan S, Bt Awang M, Abdullah MAH Malignant transformation of plexiform neurofibroma due to neglected giant soft tissue swelling of the back: a case report. Cureus [Internet] 2024. Available from: https://www.cureus.com/articles/268381-malignant-transformation-of-plexiform-neurofibroma-due-to-neglected-giant-soft-tissue-swelling-of-the-back-a-case-report. pp1-8. 10.7759/cureus.63807 PMC11297569 39099914

